# Equivalent model optimization with cyclic correction approximation method considering parasitic effect for thermoelectric coolers

**DOI:** 10.1038/s41598-017-16261-0

**Published:** 2017-11-21

**Authors:** Ning Wang, Jiajun Chen, Kun Zhang, Mingming Chen, Hongzhi Jia

**Affiliations:** Engineering Research Center of Optical Instrument and System, Ministry of Education, Shanghai Key Lab of Modern Optical System, University of Shanghai for Science and Technology, Shanghai, China

## Abstract

As thermoelectric coolers (TECs) have become highly integrated in high-heat-flux chips and high-power devices, the parasitic effect between component layers has become increasingly obvious. In this paper, a cyclic correction method for the TEC model is proposed using the equivalent parameters of the proposed simplified model, which were refined from the intrinsic parameters and parasitic thermal conductance. The results show that the simplified model agrees well with the data of a commercial TEC under different heat loads. Furthermore, the temperature difference of the simplified model is closer to the experimental data than the conventional model and the model containing parasitic thermal conductance at large heat loads. The average errors in the temperature difference between the proposed simplified model and the experimental data are no more than 1.6 K, and the error is only 0.13 K when the absorbed heat power *Q*_*c*_ is equal to 80% of the maximum achievable absorbed heat power *Q*_*max*_. The proposed method and model provide a more accurate solution for integrated TECs that are small in size.

## Introduction

With the development of semiconductor technology and packaging technology, the thermal management of electronic components has become an important factor restricting miniaturization and integration^1^. Thus, it is critical to solve thermal-induced issues to obtain a higher integration density and the better performance of on-chip systems^2^. Since solid-state cooling devices, such as TECs, are small, reliable, environmentally friendly, maintenance free, and easy to control^3^, they can be used for the thermal control of power electronics and optoelectronic components, specifically, power amplifiers, microprocessors, pump lasers and laser diodes^4^. Labudovic *et al*. applied TECs to a pump laser module for thermal management^5^. Furthermore, enhanced cooling models based on TECs have been developed to meet the thermal demand of high-power light-emitting diode (LED) headlights^6^. Still in the field of high-power LEDs, Li *et al*. effectively reduced the thermal resistance by employing TECs^7^. The performance and reliability of these components are affected by the thermal dissipation, the output light properties, for instance, the centre wavelength, spectrum, and power magnitude, of which decrease drastically with increasing component junction temperature. Hence, it is necessary to optimize the model for thermoelectric modules (TEMs) to better analyse refrigerating systems with TEMs for thermal management.

TECs are mainly composed of *p*- and *n*-type thermoelectric materials, copper conductors and ceramic plates. Many kinds of thermoelectric materials have been developed with a high thermoelectric figure of merit, ZT, in different temperature ranges^8^. The Bi_2_Te_3_ system, commonly used in low-temperature environments, is presently recognized as the most suitable thermoelectric material. Consequently, Bi_2_Te_3_-based thermoelectric materials have well-established applications in the refrigeration field. However, the ZT value of thermoelectric devices only reaches 1 at room temperature. To increase ZT, significant progress has been made in recent years using nanostructured materials, such as thin-film superlattices, thick films of quantum-dot superlattices and nanocomposites^9–11^. For example, Venkatasubramanian R *et al*. recently reported extremely high ZT values of 2.4 in p-type Bi_2_Te_3_/Sb_2_Te_3_ superlattices and 1.4 in n-type Bi_2_Te_3_/Bi_2_Te_2.83_Se_0.17_ superlattices, and this enhancement was achieved by controlling phonon and electron transport in the superlattices^12^. A maximum cooling flux of 258 W·cm^−2^ can be achieved in thermoelectric modules composed of thin-film Bi_2_Te_3_-based superlattices; nevertheless, the parasitic thermal resistance of the device reaches (3.08 ± 1.98) K/W^9^. In the optimization of the ZT value using nanostructured materials, such as thin-film superlattices^13^, thick films of quantum-dot superlattices and nanocomposites, researchers also focus on the performance characteristics of TECs. To achieve high cooling power, one serious problem is the electrical and thermal contact resistance between the metal electrodes and the thermoelectric elements, especially for Bi_2_Te_3_-based materials with low intrinsic electrical resistivity^14,15^. Since losses in Δ*T* due to intercascade thermal resistance effects are essentially higher than those related to the electrical contact resistance for alumina ceramics, the effect of the thermal contact resistance should be emphasized^16^. Thermal contact resistance is an important parasitic parameter when thermoelectric elements are short in length, whereas it is usually omitted in both theory and experiment for conventional designs. Thus, the effective ZT of the device is significantly smaller than the intrinsic ZT of the material^17^. Chávez, J.A. *et al*. considered the complete electrical and thermal behaviour of the proposed simulation circuit model for TECs with simulation program with integrated circuit emphasis (SPICE), but neglected the parasitic thermal resistance^18^. G. E. Bulman *et al*. considered the hot side of the parasitic thermal resistance, but neglected the cold end of the heat loss. As a result, there is no difference between the cold side of the internal and external temperature^10^. Moreover, X.C. Xuan investigated the effect of the thermal contact resistance of TECs with relatively short thermoelectric elements by assuming that the thermoelectric arms packing density and the ratio of the thermal conductivity and thermal contact conductivity approach the real value^19^. Furthermore, M Sim *et al*. presented both modelling and a method for extracting the parasitic thermal conductance and intrinsic parameters of TEMs based on information readily available from vendor datasheets^20^. The results of the containing parasitic thermal conductance *K*_*c*_ model are comparable with the vendor data within the current range of 1.36 A to 3.4 A, and the model does not describe the relationship between the intrinsic parameters and the parasitic thermal conductance.

In this paper, a simplified equivalent model is proposed, which is extracted from the model containing *K*_*c*_, on the basis of the conventional TEC model. The demonstrated method of cyclic correction is summarized by the use of the parameter extraction processes of the conventional model, the model containing *K*_*c*_ and the simplified equivalent model. Through the extrinsic parameters (maximum achievable temperature difference Δ*T*_*max*_, hot-side temperature *T*_*h*_, maximum input current *I*_*max*_, maximum input voltage *V*_*max*_ and maximum achievable absorbed heat power *Q*_*max*_) provided by the manufacturer and the device parameters (overall Seebeck coefficient *α*_*mC*_, overall thermal conductance *K*_*mC*_ and overall electric resistance *R*_*mC*_) obtained by the conventional extraction method, the intrinsic parameters (intrinsic Seebeck coefficient *α*_*m*_, intrinsic thermal conductance *K*_*m*_ and intrinsic electric resistance *R*_*m*_) and the parasitic parameter *K*_*c*_ can be iteratively calculated. The equivalent parameters (equivalent Seebeck coefficient *α*_*eqv*_, electric resistance *R*_*eqv*_, and thermal conductance *K*_*eqv*_) are generalized by *α*_*m*_, *K*_*m*_, *R*_*m*_ and *K*_*c*_ when the simplified equivalent model is refined from the model containing *K*_*c*_. Finally, the temperature difference comparisons between the simplified model and the experimental data verify the feasibility of the proposed method and model.

## Results and Discussion

### Cyclic correction of the TEC model

**Figure 1:**
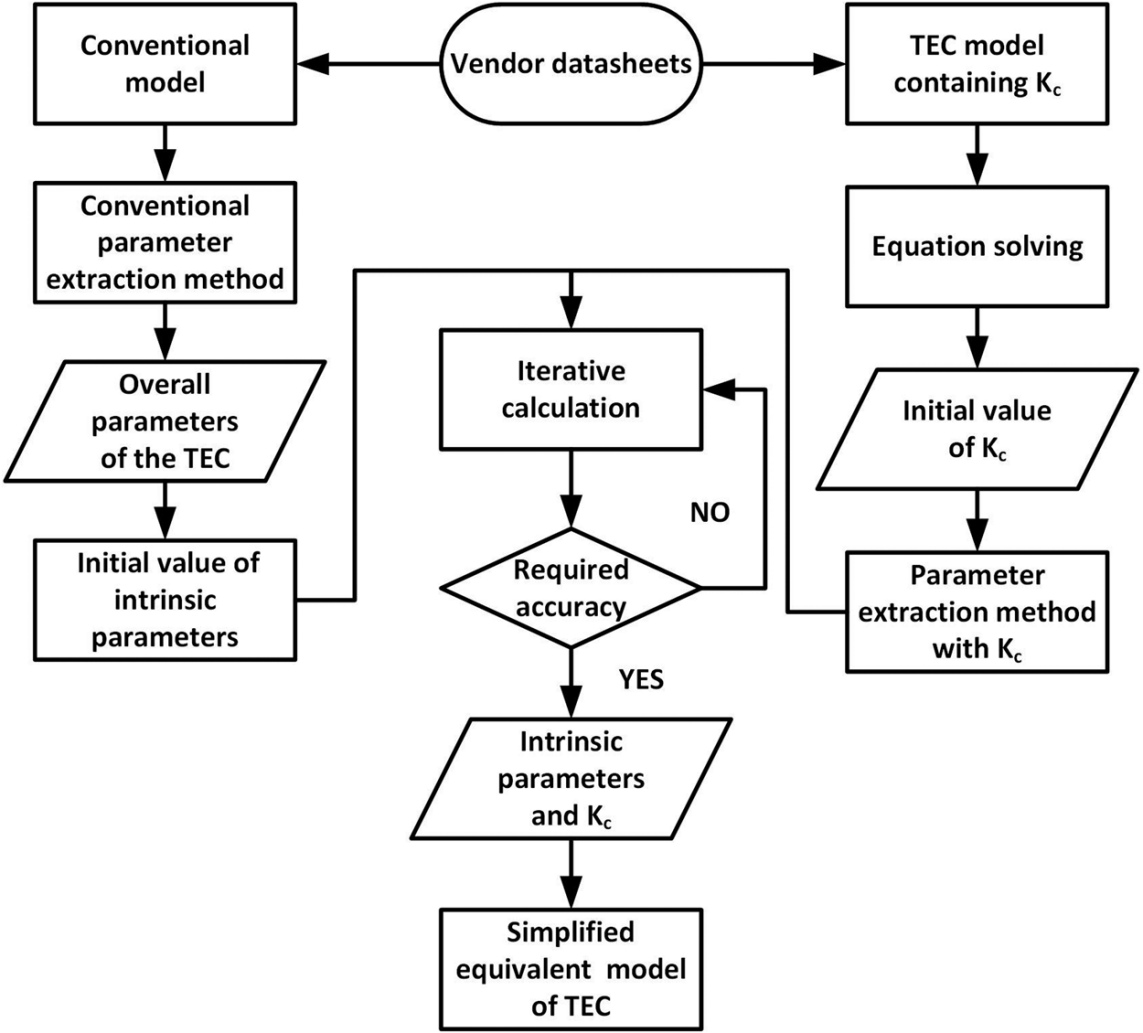
Equivalent TEC parameter evaluation methods.

The TEC model has been modified several times, but the theoretical value still has a large error compared to the actual experimental data, especially under heavy-load conditions. In the process of optimizing previous models, we combine the above models and use the cyclic correction method to evaluate the equivalent parameters for a TEC to provide a more accurate TEC model. The specific process of this method is shown in Fig. 1.

**Figure 2:**
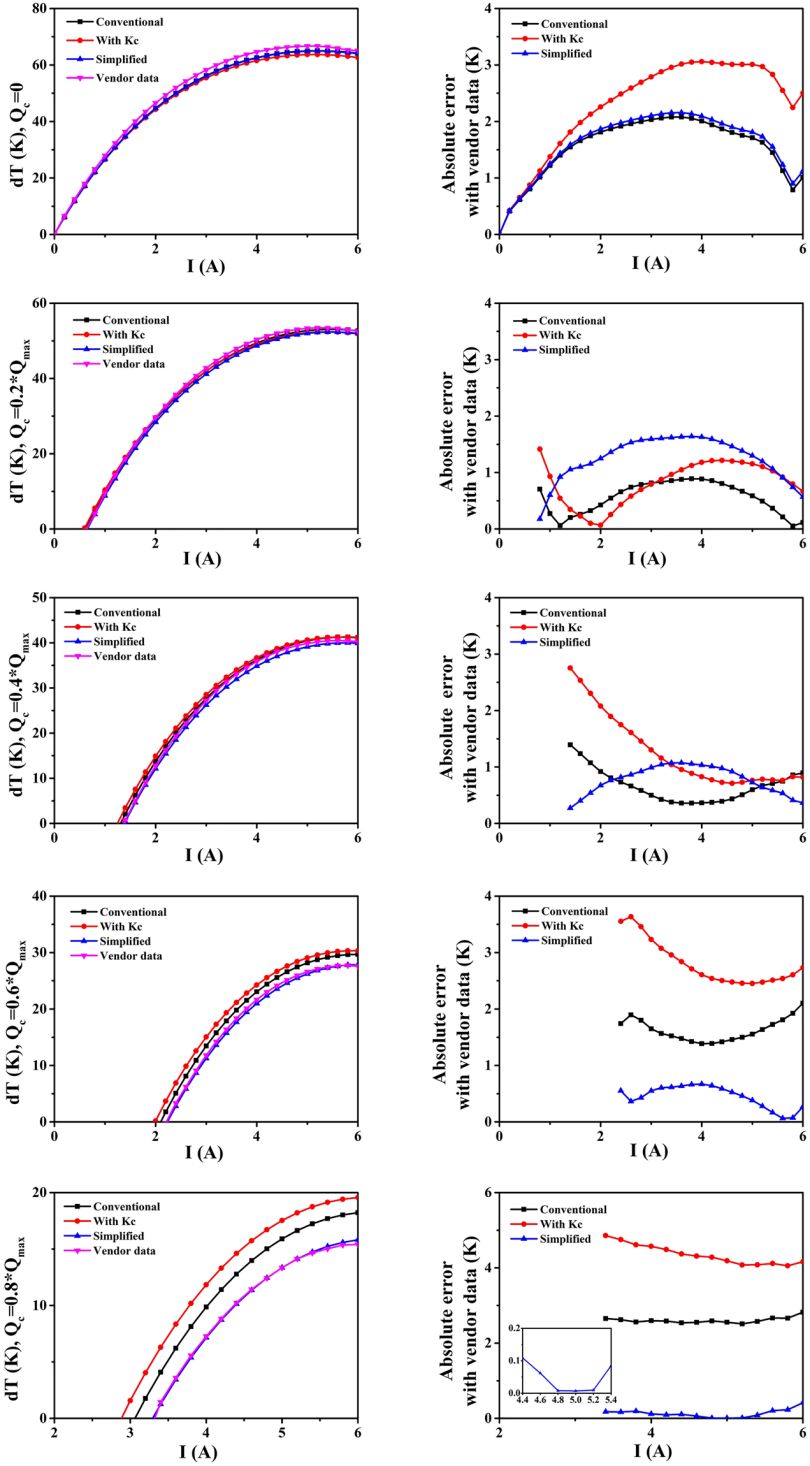
(**a**,**c**,**e**,**g**,**i**) Temperature difference Δ*T* under the conditions *Q*_*c*_ = 0, 0.2 × *Q*_*max*_, 0.4 × *Q*_*max*_, 0.6 × *Q*_*max*_, and 0.8 × *Q*_*max*_. (**b**,**d**,**f**,**h**,**j**) The corresponding absolute errors compared with the vendor data.

Through the above-described method, a simplified TEC model with equivalent parameters can be obtained, which produces results comparable to the experimental data. Temperature versus current profiles are produced from the conventional model, the model containing *K*_*c*_ and the proposed simplified equivalent model with different heat loads. Ideally, the heat load is equivalent to the absorbed heat power at the cold side. Figure 2(a,c,e,g and i) demonstrate a comparison of the temperature difference determined from all models and the experimental data under the conditions *Q*_*c*_ = 0, 0.2 × *Q*_*max*_, 0.4 × *Q*_*max*_, 0.6 × *Q*_*max*_, and 0.8 × *Q*_*max*_, respectively. In addition, Fig. 2(b,d,f,h and j) show the corresponding absolute errors compared with the experimental data.

When the current grows with a constant *Q*_*c*_, the temperature differences obtained by all models increase with the same trend as the experimental data. Figure 2(b,d,f,h and j) clearly show the proximity between each model and the vendor data. As the load increases, the temperature difference of the proposed simplified model approaches the experimental data. When the thermal load is 0.8 × *Q*_*max*_, the average absolute error is 0.13 K, and the maximum absolute error is within 0.2 K. By contrast, the average error of the conventional model is 2.61 K, and that of the model with *K*_*c*_ reaches 4.35 K.

As shown in Table 1, the relative error of simplified equivalent model is much smaller than the other models when *Q*_*c*_ = 0.8 × *Q*_*max*_. As the current increases to 4 A, 5 A and 6 A, the relative errors are within 3%, which reflects the advantages of the proposed simplified model. Moreover, the performance of the model containing *K*_*c*_ is worse than that of the conventional model since the relationships between the parasitic thermal conductivity and the other parameters are neglected. The proposed simplified model applies the equivalent parameter evaluation method to integrate the intrinsic and parasitic parameters, and therefore, the obtained temperature difference is closer to the vendor data.

**Table 1:** Relative errors of all models compared with the experimental data.

Current	4 A	5 A	6 A
Conventional model (%)	35.71	19.16	18.32
Model containing *K*_*c*_ (%)	62.90	31.37	27.02
Simplified equivalent model (%)	1.65	0.05	2.70

Notably, as an application device, the performance of a TEC under no or small load is of no practical significance. In the experiment, we were more concerned with the cooling capacity and the cold-side temperature under a certain heat load and electric power. Based on the above results, the result of the proposed simplified model is closer to the measured vendor data, with an average absolute error of 1.6 K. Therefore, the equivalent parameters obtained from the circular correction method are satisfactory. The simplified equivalent model can be applied to the theoretical analysis of temperature control for applications such as LED light sources, high-power devices, and circuits with high heat flux.

In this paper, a cyclic correction method for the TEC model is proposed with a simplified equivalent model by integrating the intrinsic parameters and parasitic thermal conductance. Because the equivalent parameters relate not only to the *K*_*c*_ but also to the current, the result of the proposed simplified model is closer to the vendor data than the conventional model and the model containing *K*_*c*_, with an average absolute error of 1.6 K. When the thermal load is 0.8 × *Q*_*max*_, the average absolute error obtained by the simplified model is 0.13 K, and its maximum relative error is within 3% compared with the measured data from the manufacturer. The accuracy of the model is also verified by the experimental results. In the case of different heat loads, the simplified model obtained by the proposed cyclic correction method accurately reproduces the performance of a commercial TEC.

## Methods

### Conventional TEC model and parameter extraction method

#### Conventional theoretical model

A typical TEC consists of many *p-n* doped thermoelectric elements sandwiched between two electrically insulated but heat-conducting ceramic plates. Four basic physical phenomena are associated with the operation of TECs: the Seebeck effect, the Peltier effect, the Thomson effect, and the Joule effect.

When an electric current flows through the TEC, the heat transfer can be determined by the following equations^19–21^:

1 $${Q}_{c}={\alpha }_{mC}{T}_{c}I-{K}_{mC}({T}_{h}-{T}_{c})-\frac{1}{2}{R}_{mC}{I}^{2}$$

2 $${Q}_{h}={\alpha }_{mC}{T}_{h}I-{K}_{mC}({T}_{h}-{T}_{c})+\frac{1}{2}{R}_{mC}{I}^{2}$$

where *Q*_*c*_ is the heat power absorbed at the cold side of the TEC; *Q*_*h*_ is the heat power released at the hot side of the TEC; *α*_*mC*_ is the overall Seebeck coefficient; *R*_*mC*_ is the overall electric resistance; *K*_*mC*_ is the overall thermal conductance; and *T*_*c*_ and *T*_*h*_ are the temperatures of the cold and hot sides of the thermoelectric device, respectively.

The electric power *P*_*e*_ can be expressed as the difference between the absorbed and released heat:

3 $${P}_{e}={Q}_{h}-{Q}_{c}={\alpha }_{mC}I({T}_{h}-{T}_{c})+{I}^{2}{R}_{mC}$$

An electric voltage is applied to the TEC to overcome the Seebeck voltage and the electric resistance:

4 $$V=P/I\,{\alpha }_{mC}({T}_{h}-{T}_{c})+I{R}_{mC}$$

when *T*_*h*_ is fixed and *Q*_*c*_ is known, the change in the temperature difference Δ*T* with current can be obtained:

5 $${\rm{\Delta }}T=\frac{{\alpha }_{mC}{T}_{h}I-\frac{1}{2}{R}_{mC}{I}^{2}-{Q}_{c}}{{\alpha }_{mC}I+{K}_{mC}}$$

#### Conventional parameter extraction method

The traditional method for calculating the TEC device parameters involves using the extrinsic performance parameters provided by the manufacturer^3^. The following extrinsic parameters are provided: Δ*T*_*max*_, maximum achievable temperature difference with the hot-side temperature *T*_*h*_; *I*_*max*_, maximum input current producing the maximum temperature difference; *V*_*max*_, maximum input voltage corresponding to the electrical current; and *Q*_*max*_, maximum achievable absorbed heat power, respectively. When the absorbed heat power reaches *Q*_*max*_, the temperature difference Δ*T* is zero.

Accordingly, when the material properties are assumed to be independent of temperature, the overall Seebeck coefficient, electric resistance, and thermal conductance of the module can be expressed as follows:

6 $${\alpha }_{mC}=\frac{{V}_{{\max }}}{{T}_{h}}$$

7 $${R}_{mC}=\frac{{V}_{max}}{{I}_{max}}(1-\frac{{\rm{\Delta }}{T}_{max}}{{T}_{h}})$$

8 $${K}_{mC}=\frac{{V}_{max}{I}_{max}({T}_{h}-{\rm{\Delta }}{T}_{max})}{2{\rm{\Delta }}{T}_{max}{T}_{h}}$$

The extracted parameters are regarded as the device parameters of conventional TECs but do not account for parasitic thermal and electrical effects. Similar to other methods for extracting the device parameters from data given by the manufacturer^8^, the extracted thermoelectric data using this traditional method have a certain error compared with the intrinsic and actual parasitic parameters for TECs.

### TEC model with parasitic thermal conductance and its parameter extraction method

#### TEC model with parasitic thermal conductance

**Figure 3:**
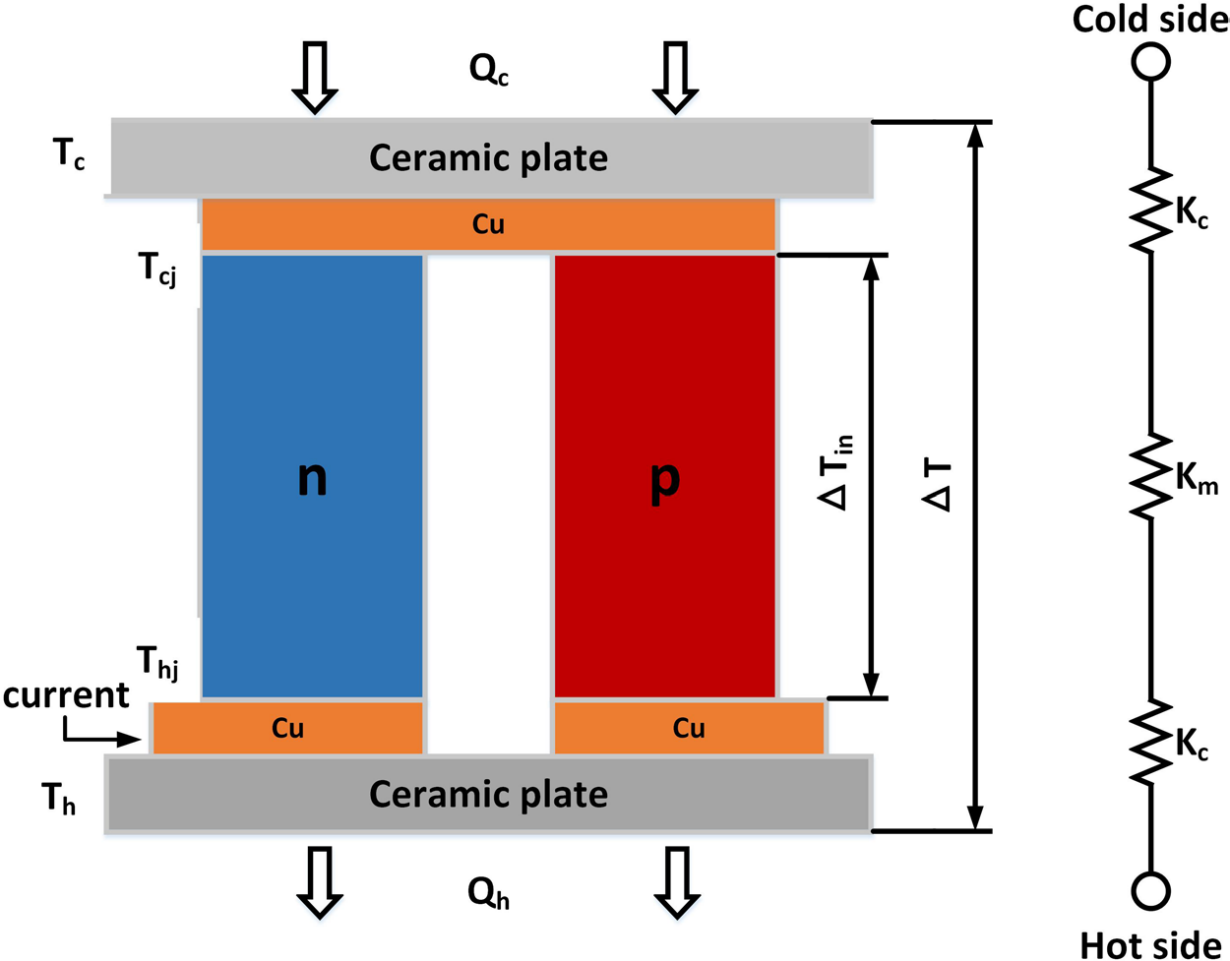
Basic configuration and thermal conductance network of a TEC.

One TE element is extracted as the analytic object for the TEC structure, as shown in Fig. 3. Relative to the conventional TEC model, the concept of parasitic thermal conductance *K*_*c*_ is proposed, which is defined as the sum of the thermal and contact thermal conductance of the ceramic plates and copper conductors on both sides of the device. Here, *K*_*c*_ at both sides are assumed to be equal due to the symmetric structure of the TEC.

Evidently, heat transfer in a TEC at each junction is different from equations (1 and 2), as follows:

9 $${Q}_{c}={K}_{c}({T}_{c}-{T}_{cj})$$

10 $${Q}_{c}={\alpha }_{m}{T}_{cj}I-{K}_{m}({T}_{hj}-{T}_{cj})-\frac{1}{2}{R}_{m}{I}^{2}$$

11 $${Q}_{h}={\alpha }_{m}{T}_{h}I-{K}_{m}({T}_{hj}-{T}_{cj})+\frac{1}{2}{R}_{m}{I}^{2}$$

12 $${Q}_{h}={K}_{c}({T}_{hj}-{T}_{h})$$

where *α*_*m*_, *K*_*m*_, and *R*_*m*_ are the intrinsic Seebeck coefficient, the thermal conductance, and the electric resistance of the module, respectively. Due to the presence of parasitic effects, the external temperature difference Δ*T* is slightly lower than the internal temperature difference Δ*T*_*in*_. The temperature difference over these intervals, (*T*_*c*_ − *T*_*cj*_) and (*T*_*hj*_ − *T*_*h*_), is given by *Q*_*c*_/*K*_*c*_ and *Q*_*h*_/*K*_*c*_. Thus, Δ*T*_*in*_ can be represented as:

13 $${\rm{\Delta }}{T}_{in}={T}_{hj}-{T}_{cj}=({T}_{hj}-{T}_{h})-({T}_{c}-{T}_{cj})+({T}_{h}-{T}_{c})=2\frac{{Q}_{c}}{{K}_{c}}+\frac{VI}{{K}_{c}}+{\rm{\Delta }}T$$

Then, substituting equation (13) into equation (10) with extrinsic parameters that are easily measurable, the absorbed heat power *Q*_*c*_ is related to *K*_*c*_ as follows:

14 $${Q}_{c}=\frac{{\alpha }_{m}{K}_{c}{T}_{c}I-{K}_{m}IV-{K}_{m}{K}_{c}{\rm{\Delta }}T-\frac{1}{2}{K}_{c}{R}_{m}{I}^{2}}{{\alpha }_{m}I+2{K}_{m}+{K}_{c}}$$

The voltage-current relationship is redefined by the intrinsic parameters:

15 $$V={\alpha }_{m}({T}_{hj}-{T}_{cj})+I{R}_{m}$$

In the form of the substitution above, the voltage *V* is related to *K*_*c*_ as follows:

16 $$V=\frac{2{\alpha }_{m}{Q}_{c}+{\alpha }_{m}{K}_{c}{\rm{\Delta }}T+{K}_{c}I{R}_{m}}{{K}_{c}-{\alpha }_{m}I}$$

Assuming that *K*_*c*_ is infinite, equations (14 and 16) can be simplified to equations (1 and 4) of the conventional model. More significantly, when equations (14 and 16) are substituted with each other, *V* and *Q*_*c*_ can be changed into new expressions in which all extrinsic parameters are provided by the manufacturer except for the unknown value of *K*_*c*_:

17 $$V=\frac{{{\alpha }_{m}}^{2}{K}_{c}({T}_{h}+{T}_{c})+{\alpha }_{m}{{K}_{c}}^{2}{\rm{\Delta }}T+{R}_{m}{K}_{c}I({K}_{c}+2{K}_{m})}{{K}_{c}({K}_{c}+2{K}_{m})-{{\alpha }_{m}}^{2}{I}^{2}}$$

18 $${Q}_{c}=\frac{{\alpha }_{m}{K}_{c}{T}_{c}I-(\frac{1}{2}{K}_{c}+\frac{{K}_{m}{K}_{c}}{{K}_{c}-{\alpha }_{m}I}){R}_{m}{I}^{2}-(1+\frac{{\alpha }_{m}I}{{K}_{c}-{\alpha }_{m}I}){K}_{m}{K}_{c}{\rm{\Delta }}T}{{\alpha }_{m}I+2{K}_{m}+{K}_{c}+\frac{2{\alpha }_{m}{K}_{m}I}{{K}_{c}-{\alpha }_{m}I}}$$

when *T*_*h*_ is fixed and *Q*_*c*_ is known, the temperature difference can be expressed as:

19 $${\rm{\Delta }}T=\frac{{\alpha }_{m}{T}_{h}I-(\frac{1}{2}+\frac{{K}_{m}{K}_{c}}{{K}_{c}-{\alpha }_{m}I}){R}_{m}{I}^{2}-{Q}_{c}}{{\alpha }_{m}I+{K}_{m}+\frac{{\alpha }_{m}{K}_{m}I}{{K}_{c}-{\alpha }_{m}I}}$$

#### Parameter extraction method with parasitic thermal conductance

To make the expressions more concise, *K*_*m*_/*K*_*c*_ is defined as *κ*, and 1 − *α*_*m*_ *I*_*max*_/*K*_*c*_ as *ξ*. The thermoelectric parameter extraction method with parasitic thermal conductance is as follows:

20 $${\alpha }_{m}=\frac{\xi (1+2\kappa ){V}_{max}}{2\kappa {\rm{\Delta }}{T}_{max}+{T}_{h}}$$

21 $${R}_{m}=\frac{\xi {V}_{max}({T}_{h}-{\rm{\Delta }}{T}_{max})}{{I}_{max}(2\kappa {\rm{\Delta }}{T}_{max}+{T}_{h})}$$

22 $${K}_{m}=\frac{\xi (1+2\kappa ){V}_{max}{I}_{max}({T}_{h}-{\rm{\Delta }}{T}_{max})}{2(2\kappa {\rm{\Delta }}{T}_{max}+{T}_{h}){\rm{\Delta }}{T}_{max}}$$

where *α*_*m*_, *K*_*m*_, and *R*_*m*_ are all known intrinsic parameters. When *T*_*c*_ = *T*_*h*_, *V*_*max*_, *I*_*max*_ and *Q*_*max*_ can be determined for the TEC. Under the condition of *Q*_*c*_ = *Q*_*max*_, equation (18) can be transformed into a quadratic equation to solve *K*_*c*_ as follows:

23 $$({\alpha }_{m}{T}_{h}{I}_{max}-\frac{1}{2}{R}_{m}{I}_{max}^{2}-{Q}_{max}){K}_{c}^{2}-(2{K}_{m}{Q}_{max}+({K}_{m}{R}_{m}+{\alpha }_{m}^{2}{T}_{h}){I}_{max}^{2}-\frac{1}{2}{\alpha }_{m}{R}_{m}{I}_{max}^{3}){K}_{c}+{\alpha }_{m}^{2}{I}_{max}^{2}{Q}_{max}=0$$

Since the intrinsic parameters and parasitic parameter *K*_*c*_ are recursively related and *K*_*c*_ is difficult to measure, they can only be obtained by iterative calculations^20^.

The extrinsic parameters provided by a commercial TEC manufactured by RMT are shown in Table 2. Using the following data, the intrinsic parameters and parasitic thermal conductance of the TEC can be iteratively calculated.

**Table 2:** TEC module datasheet (1MC06-126-03).

Type	1MC06-126-03
Hot-side temperature *T*_*h*_ (*K*)	300
Maximum absorbed heat power *Q*_*max*_ (*W*)	45.4
Maximum temperature difference Δ*T*_*max*_ (*K*)	65
Maximum voltage *V*_*max*_ (*V*)	15.5
Maximum current *I*_*max*_ (*A)*	5.1
Measured module resistance *R*_*e*_ (*Ω*)	2.18

#### The method for the recursive iterative calculation is as follows

1. Using equations (6–8) and the extrinsic data from the manufacturer, set *α*_*mC*_, *K*_*mC*_, and *R*_*mC*_ as the initial values of *α*_*m*_, *K*_*m*_, and *R*_*m*_ based on the previously obtained results.
2. Calculate *K*_*c*_ by equation (23) using the initial values from step (1).
3. Substitute *K*_*c*_ into equations (20–22) and recalculate more accurate values of *α*_*m*_, *K*_*m*_, and *R*_*m*_.
4. Recalculate *K*_*c*_ by equation (23) using the values of *α*_*m*_, *K*_*m*_, and *R*_*m*_ from step (3).
5. Repeat steps (3) and (4) until each parameter stabilizes to the required accuracy.

**Figure 4:**
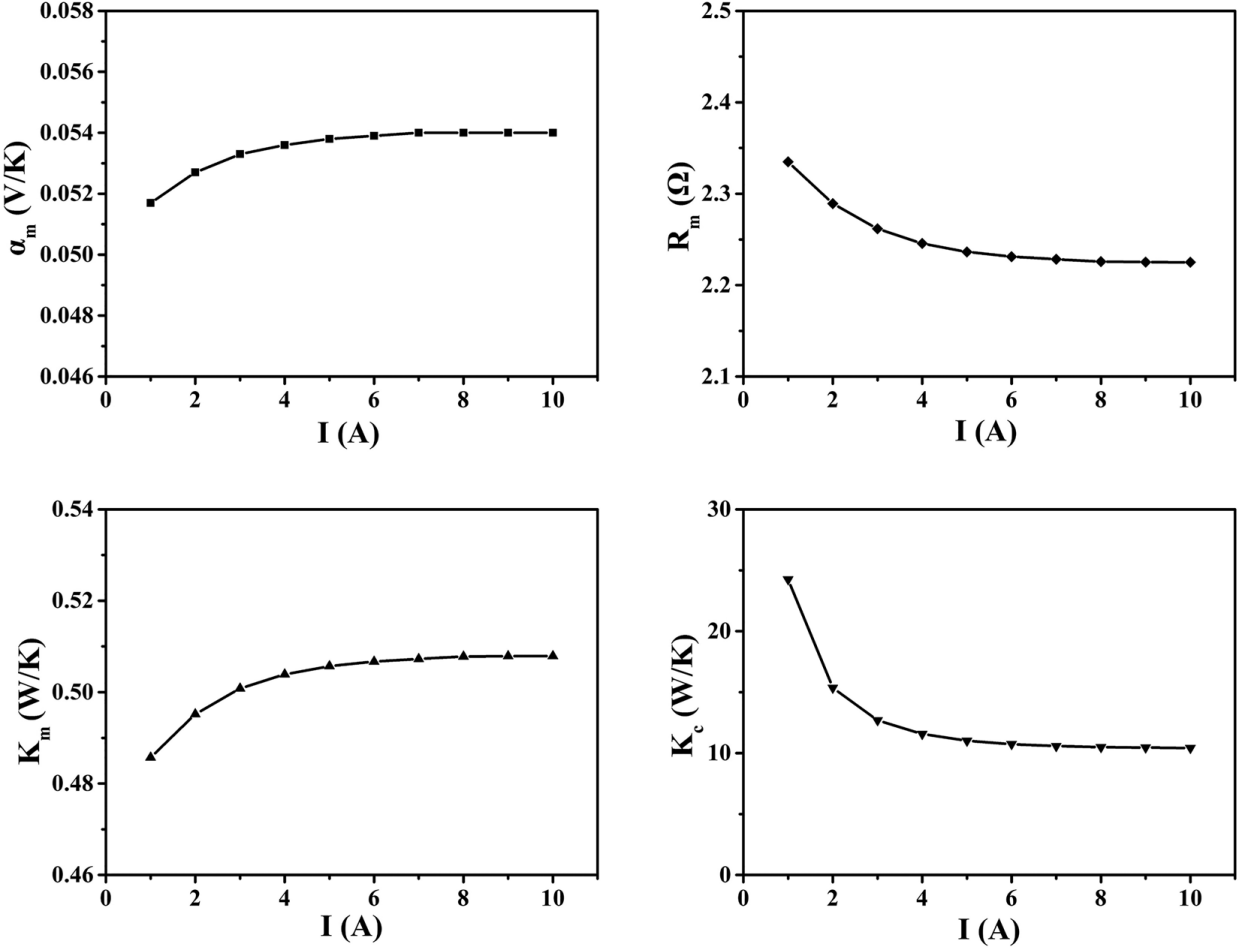
(**a**,**b**,**c**) Intrinsic parameters and (**d**) parasitic thermal conductance.

Figure 4 shows that the parameters slowly break their relationship with *K*_*c*_ and approach the intrinsic values in the iterative process. After the tenth iteration, the values of the intrinsic parameters and parasitic thermal conductance are converged within 1% errors. In general, *α*_*m*_, *K*_*m*_, *R*_*m*_, and *K*_*c*_ stabilize at 0.0540 V/K, 2.2250 Ω, 0.5079 W/K and 10.4212 W/K, respectively.

### Simplified equivalent model for thermoelectric parameters

As a result of the introduction of equivalent parameters, general formulae are developed to take into account the parasitic effects, which are more concise than the model containing *K*_*c*_. The equivalent parameters – the equivalent Seebeck coefficient *α*_*eqv*_, the electric resistance *R*_*eqv*_, and the thermal conductance *K*_*eqv*_ – in relation to *K*_*c*_ can be described as^19^:

24 $${\alpha }_{eqv}={\alpha }_{m}\frac{1-I{\alpha }_{m}/{K}_{c}}{1+2{K}_{m}/{K}_{c}-{(I{\alpha }_{m}/{K}_{c})}^{2}}$$

25 $${R}_{eqv}={R}_{m}\frac{1+2{K}_{m}/{K}_{c}-I{\alpha }_{m}/{K}_{c}}{1+2{K}_{m}/{K}_{c}-{(I{\alpha }_{m}/{K}_{c})}^{2}}$$

26 $${K}_{eqv}={K}_{m}\frac{1}{1+2{K}_{m}/{K}_{c}-{(I{\alpha }_{m}/{K}_{c})}^{2}}$$

Finally, the equations concerning *Q*_*c*_ and Δ*T* can be simplified as:

27 $${Q}_{c}={\alpha }_{eqv}{T}_{c}I-{K}_{eqv}({T}_{h}-{T}_{c})-\frac{1}{2}{R}_{eqv}{I}^{2}$$

28 $${\rm{\Delta }}T=\frac{{\alpha }_{eqv}{T}_{h}I-\frac{1}{2}{R}_{eqv}{I}^{2}-{Q}_{c}}{{\alpha }_{eqv}I+{K}_{eqv}}$$

Because *K*_*c*_ is related to many intrinsic parameters, it is not only cumbersome to use *Q*_*c*_ and Δ*T* to express the formula with *K*_*c*_, but this method also has some deficiencies. Using the intrinsic parameters from the above model, equations (17 and 18) can be transformed into the simple form of equations (27 and 28) by the substitution of equations (24–26). Meanwhile, *α*_*m*_, *K*_*m*_ and *R*_*m*_ are converted into *α*_*eqv*_, *R*_*eqv*_ and *K*_*eqv*_, respectively.

Because *α*_*m*_ ≪ *K*_*c*_ (that is, (*Iα*_*m*_/*K*_*c*_)^2^  ≪ 1), the parameters above can be simplified as follows:

29 $${\alpha }_{eqv}=\frac{{\alpha }_{m}(1-I{\alpha }_{m}/{K}_{c})}{1+2{K}_{m}/{K}_{c}}$$

30 $${R}_{eqv}={R}_{m}\frac{1+2{K}_{m}/{K}_{c}-I{\alpha }_{m}/{K}_{c}}{1+2{K}_{m}/{K}_{c}}$$

31 $${K}_{eqv}=\frac{{K}_{m}}{1+2{K}_{m}/{K}_{c}}$$

where *α*_*eqv*_, *R*_*eqv*_ and *K*_*eqv*_ are closely related to *K*_*c*_ and *I*. More significantly, *α*_*eqv*_ and *R*_*eqv*_ will change linearly, rather than staying a fixed value, with an increase in current.

**Figure 5:**
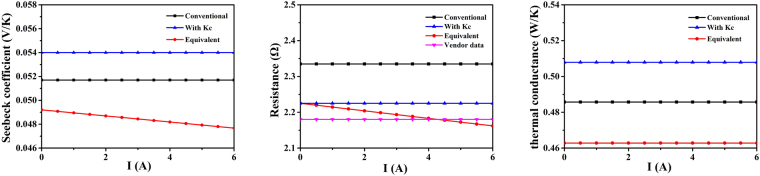
Comparison of the parameters for all models: (**a**) Seebeck coefficient, (**b**) resistance, and (**c**) thermal conductance.

As seen from Fig. 5(a), the intrinsic Seebeck coefficient *α*_*m*_ is larger than the overall Seebeck coefficient *α*_*mC*_ obtained from the conventional model as well as the equivalent Seebeck coefficient *α*_*eqv*_ obtained from the simplified equivalent model. It is because of *K*_*c*_ and the current that *α*_*eqv*_ is much smaller. In Fig. 5(b), the resistance *R*_*m*_ calculated with *K*_*c*_ is closer to the vendor data than the resistance *R*_*mC*_ calculated by the conventional model. Moreover, in comparison to the model containing *K*_*c*_, the equivalent resistance *R*_*eqv*_ is closer to the experimental data from the manufacturer, which is consistent with the vendor data at *I* = 4.2 A. As shown in Fig. 5(c), the intrinsic thermal conductance *K*_*m*_ is larger than the conventional thermal conductance *K*_*mC*_ and equivalent thermal conductance *K*_*eqv*_. Considering the cause of the parasitic thermal conductance, *K*_*eqv*_ is much smaller than the others.

By substituting equations (30 and 31) into equations (27–29), the equations can be expressed by the intrinsic and parasitic parameters:

32 $${Q}_{c}=\frac{{\alpha }_{m}{T}_{c}I(1-I{\alpha }_{m}/{K}_{c})}{1+2{K}_{m}/{K}_{c}}-\frac{{K}_{m}({T}_{h}-{T}_{c})}{1+2{K}_{m}/{K}_{c}}-\frac{{R}_{m}{I}^{2}(1+2{K}_{m}/{K}_{c}-I{\alpha }_{m}/{K}_{c})}{2+4{K}_{m}/{K}_{c}}$$

33 $${\rm{\Delta }}T=\frac{{\alpha }_{m}{T}_{h}I(1-I{\alpha }_{m}/{K}_{c})-\frac{1}{2}{R}_{m}{I}^{2}(1+2{K}_{m}/{K}_{c}-I{\alpha }_{m}/{K}_{c})-{Q}_{c}(1+2{K}_{m}/{K}_{c})}{{\alpha }_{m}I(1-I{\alpha }_{m}/{K}_{c})+{K}_{m}}$$
